# Clinical breast examination and its associated factors among reproductive age women in Ghana: multilevel logistic regression analysis

**DOI:** 10.3389/fonc.2024.1413076

**Published:** 2024-12-24

**Authors:** Zenebe Abebe Gebreegziabher, Birhan Ewunu Semagn, Agmasie Damtew Walle, Mahider Awoke Belay, Wubet Tazeb Wondie, Gezahagn Demsu Degefaw, Werkneh Melkie Tilahun, Ermiyas Endewunet Melaku, Tadesse Mamo Dejene

**Affiliations:** ^1^ Department of Epidemiology and Biostatistics, School of Public Health, Debre Berhan University, Debre Berhan, Ethiopia; ^2^ Department of Public Health, School of Public Health, Debre Berhan University, Debre Berhan, Ethiopia; ^3^ Department of Public Health, College of Medicine and Health Science, Injibara University, Injibara, Ethiopia; ^4^ Department of Pediatrics and Child Health Nursing, College of Medicine, and Health Science, Ambo University, Ambo, Ethiopia; ^5^ Department of Neonatal Health Nursing, School of Nursing, College of Medicine and Health Sciences, and Specialized Hospital, University of Gondar, Gondar, Ethiopia; ^6^ Department of Public Health, College of Medicine and Health Sciences, Debre Markos University, Debre Markos, Ethiopia; ^7^ Department of Internal Medicine, School of Medicine, Debre Berhan University, Debre Berhan, Ethiopia

**Keywords:** clinical breast examination, GDHS, reproductive age women, multilevel, Ghana

## Abstract

**Background:**

Breast cancer poses a significant health burden in Ghana and globally, being the primary cause of cancer-related illness and death among women. The World Health Organization has identified clinical breast examination as the gold standard for women in low and middle-income countries. However, the uptake of clinical breast examination remains low in these settings, including Ghana, where the nationwide prevalence and associated factors of this practice have not been determined. Therefore, this study aimed to assess the prevalence and factors associated with clinical breast examination among women of reproductive age in Ghana, using data from 2022 Ghanaian Demographic and Health Survey.

**Methods:**

In this study, data from the most recent Ghanaian Demographic Health Survey conducted in 2022 were utilized. The survey used a two-stage stratified sampling technique, and a weighted sample of 15,013 participants was included in the analysis. Descriptive statistics, such as frequencies, percentages, and graphical representations, were utilized to present the study’s findings. Multilevel mixed-effects logistic regression analysis was employed to identify factors associated with clinical breast examination.

**Results:**

The study found that the prevalence of clinical breast examination was 18.39% (95% CI: 17.8-19.0%). Age group of 45 to 49 (AOR=2.84, 95% CI: 2.13, 3.78), having completed secondary education (AOR=1.70, 95% CI: 1.41, 2.06), having diploma or above education (AOR=3.63, 95% CI: 2.86, 4.61), using modern contraception (AOR=1.12, 95% CI: 1.00, 1.25), having health insurance coverage (AOR=1.53, 95% CI: 1.24, 1.89), listening to the radio at least once per week (AOR=1.35, 95% CI: 1.20, 1.53), reading a newspaper at least once per week (AOR=1.75, 95% CI: 1.39, 2.21), being tested for HIV (AOR=1.92, 95% CI: 1.68, 2.19), undergoing screening for cervical cancer (AOR=6.64, 95% CI: 5.51, 7.99), being currently employed (AOR=1.17, 95% CI: 1.02, 1.34), visiting a health facility within the past 12 months (AOR=1.36, 95% CI: 1.23, 1.51), belonging to the wealthiest wealth categories (AOR=1.70, 95% CI: 1.27, 2.28), being from the North East region (AOR=1.96, 95% CI: 1.19, 3.22) or Oti region (AOR=0.54, 95% CI: 0.34, 0.92), having a greater distance to a health facility (AOR=0.86, 95% CI: 0.75, 0.98), and being from a community with a higher proportion of educated individuals (AOR=1.31, 95% CI: 1.07, 1.61) were significant associated factors of clinical breast examination.

**Conclusions and recommendations:**

The study revealed that the magnitude of clinical breast examination among Ghanaian women was low. Age, educational status, modern contraceptives utilization, health insurance coverage, media exposure, HIV testing, cervical cancer screening, occupation, health facility visits, wealth index, and region were significantly associated with clinical breast examination. These findings suggest that public health interventions should prioritize addressing these factors to increase clinical breast examination uptake and promote early detection of breast cancer to improve the survival of women with breast cancer.

## Background

A 2019 WHO report identified cancer as a leading cause of death in 112 out of 183 countries (1), with breast cancer being the most prevalent among women. In 2020, 2.3 million new cases of breast cancer were diagnosed, leading to 685,000 deaths (2). Globally, breast cancer mortality rates are rising, causing 700,660 deaths and 20.6 million disability-adjusted life years (DALYs) in 2019 (3–5). In sub-Saharan Africa (SSA), breast cancer is the leading cause of cancer-related deaths, with cases expected to double by 2040 due to aging populations (6). Unlike high-income countries, where survival rates are improving, SSA faces low survival rates, with around 50% of women surviving less than five years due to late-stage diagnoses and inadequate healthcare (6–10). In Ghana, breast cancer is the leading cause of cancer deaths, with 2,900 new cases annually (11, 12). According to a report by Ghana’s Ministry of Health (MOH), around 80% of diagnoses occur at advanced stages, resulting in a three-year survival rate of 52%, highlighting the need for better healthcare infrastructure and early detection strategies (13, 14).

The screening or early detection of breast cancer, particularly during asymptomatic or pre-clinical stages, offers significant advantages in the treatment of patients. Previous studies have provided evidence that screening has a substantial impact on the timing of mortality in breast cancer cases. Specifically, patients with a history of early detection or screening of breast cancer have shown a reduction in mortality rates by up to 41 percent within a ten-year timeframe. This highlights the crucial role of screening in improving outcomes and reducing the impact of breast cancer on patient health (15–17). The commonly utilized screening methods for breast cancer include mammography, breast self-examination, and clinical breast examination. The WHO advocates for population-based mammography as the preferred and superior screening method for breast cancer, with the potential to decrease breast cancer mortality by 20-35% (18). However, in developing countries like Ghana, the use of mammography as a screening method is hampered by cost and limited availability. Therefore, clinical breast examination(CBE) is considered a viable alternative for breast cancer screening in such settings (19). Several studies indicate that CBE could be more successful than mammography in detecting breast cancer among younger women and individuals with dense breast tissue (20–22). There was evidence to suggest that if a CBE had not been carried out, a sizable number of malignancies would have gone undiscovered. Clinical breast examination is a relatively inexpensive diagnostic that may help identify breast cancer and may be used to order breast ultrasonography if a mammogram is negative (23, 24).

The WHO and American Cancer Society recommend that women aged 25 to 40 undergo clinical breast examination every 3 to 5 years, and women aged 40 and above should have it annually. Although the primary focus is on women aged 25 and above, it is worth noting that breast cancer can be detected as early as age 20, and it can impact women of any age. Extending screening to encompass all women of reproductive age may provide advantages (25, 26). In the current study, we included all women within the reproductive age range for comprehensive coverage.

Despite the significance of clinical breast examination and the recommendations by the World Health Organization (WHO) and American Cancer Society (ACS) for its implementation among all women, the utilization of clinical breast examination remains low in various countries. In Sub-Saharan Africa, the proportion of clinical breast examinations ranges from 0.9% in Tanzania to 53.3% in Malaysia (27–33). According to various reports and studies, factors such as income, place of residence, sex of household head, current pregnancy status, distance from health facilities, age, level of education, utilization of modern contraceptives, health insurance coverage, frequency of listening to the radio, frequency of reading newspapers or magazines, previous HIV testing, cervical cancer testing, occupation, awareness of breast cancer, visits to a health facility within the past 12 months, and region have been identified as significant variables (27–29, 34–38).

For the design, planning, and implementation of effective and sustainable interventions to improve the uptake of clinical breast examination (CBE) for early detection of breast cancer and to enhance the survival of patients, understanding the determinants of CBE uptake within a specific context is essential. However, to our knowledge, no study has assessed the prevalence of and factors associated with CBE in Ghana at national level, apart from local studies conducted among future healthcare professionals in the country (39), in Nandom Municipality (40) and among older women (41). Therefore, this study aimed to examine the current prevalence of CBE and identify the factors associated with it among reproductive-age women in Ghana using data from the Ghanaian Demographic and Health Survey 2022 (GDHS, 2022).

## Methods and materials

### Data source and participants

The study utilized data from the most recent Demographic and Health Survey of Ghana (GDHS, 2022), which is the seventh survey conducted by the Ghana Statistical Service (GSS) in collaboration with the Ministry of Health/Ghana Health Service (MoH/GHS) and other stakeholders. The Demographic and Health Survey (DHS) is a community-based cross-sectional survey aimed at providing up-to-date information on the health and related conditions of the community. The survey covered a broad spectrum of data, including topics such as fertility preferences, awareness and usage of family planning methods, maternal and child health indicators, knowledge and attitudes towards HIV/AIDS and other sexually transmitted infections (STIs), and the prevalence of HIV among adults. It was conducted across all sixteen regions of Ghana and involved different types of records, such as records for men, reproductive-age women, births, and birth cohorts. In this study, individual records were specifically used from a weighted sample of 15,013 individuals selected from a total of 618 clusters. A two-stage stratified sampling technique was utilized in this survey. The sampling process involved selecting samples from the sampling frame in two stages. The regions were divided into urban and rural areas, and independent samples were chosen within each stratum using a two-stage selection method. In the first stage, 618 Enumeration Areas (EAs) were selected based on their size using a probability proportional to size approach. In the second stage, a fixed number of 30 households were selected through equal probability systematic sampling in both urban and rural clusters. The detailed methodology is available in the survey report (42).

### Variables of the study

#### Outcome variable

The outcome variable for this study was clinical breast examination. Participants were specifically asked if a healthcare provider had examined their breasts for breast cancer, including mammographic and ultrasound examinations. Those who answered yes were coded as ‘1,’ indicating that they had undergone a clinical breast examination. On the other hand, participants who responded with ‘no’ were categorized as ‘0,’ indicating that they had not received a clinical breast examination. There was a single respondent who said ‘I did not know,’ and this observation was omitted.

#### Independent variables

This study investigated a total of 22 explanatory variables obtained from various literature sources (28, 29, 36, 43, 44). The variables were selected based on their availability in the dataset. Out of these, 16 variables pertained to individual characteristics, while the remaining six variables were related to community-level factors. The individual-level variables include age, marital status, education level, current pregnancy status, frequency of television watching, radio listening, and reading newspapers or magazines, history of HIV and cervical cancer testing, current employment status, wealth index, recent healthcare facility visits within the past 12 months, sex of the household head, and coverage by health insurance. These variables were directly utilized as presented in the GDHS dataset. Regarding modern contraceptive utilization, the information was derived from the available dataset. Respondents who reported using methods such as condoms, pills, injectables, implants, intrauterine devices, or sterilization were classified as modern contraceptive users, while those who used other methods or did not use any method were categorized as non-users of modern contraceptives. The parity variable, which indicates the number of children a woman has given birth to, was re-categorized into three groups: 0, 1-3, and 4 or more. In the GDHS dataset, community-level variables were not directly collected, except for residence, region, and distance to health facility. To address this, we created community-level variables by aggregating data at the enumeration area level. Specifically, we generated community wealth, community education, and community health insurance coverage. Since the data for these variables did not follow a normal distribution, we used the national median value as a threshold for categorization. Communities with values equal to or above the median were classified as having high community wealth, high community education, and high community health insurance coverage. Conversely, communities with values below the median were categorized as having low community wealth, low community education, and low community health insurance coverage.

### Data management and statistical analysis

Data management and statistical analysis were performed using STATA version 16. The descriptive and inferential statistics result was based on the weighted sample using V005/1000000. Descriptive results were presented using frequency, percentage, and graph. For the inferential statistics chi-square test, bivariable and multivariable mixed effect multilevel logistic regression analysis were conducted. In the chi-square test, which examined the association between clinical breast examination and other categorical variables, variables with a p-value less than 0.05 were deemed to have a statistically significant association. These variables were then considered for bivariable analysis. In the bivariable analysis, variables with a p-value below 0.2 were selected for inclusion in the multivariable analysis. In the multivariable model, variables with a p-value below 0.05 were considered to have a statistically significant association. The strength of the relationship between the dependent and independent variables was assessed using Adjusted Odds Ratio (AOR) accompanied by a 95% Confidence Interval (CI). To account for the hierarchical structure of the DHS data and the relatively high intra-class correlation value (17%) (45), a mixed-effects multilevel model was applied. The outcome variable focused on the binary nature of clinical breast examination, indicating its presence or absence. Thus, a mixed-effects multilevel binary logistic regression model was employed. The data underwent modeling using four distinct multilevel mixed-effects logistic regression models. These models encompassed the null model (lacking independent variables), the individual variable-only model (involving solely individual-level variables), the community variable model (including solely community-level variables), and the full model (incorporating both individual and community-level variables). Given the hierarchical structure of the DHS data, women within the same cluster may share similar characteristics, unlike those from different clusters. This can violate traditional regression model assumptions, particularly the independence of observations and equal variance. To address this, a multilevel model was used to investigate factors associated with CBE, with Enumeration Area (V001) included as a random variable. The Intraclass Correlation Coefficient (ICC) and Median Odds Ratio (MOR) were computed to assess the variation between clusters. To compare the performance of these models, deviance, the Akaike Information Criterion (AIC), and the Bayesian Information Criterion (BIC) were used. The full model, which incorporated both individual and community-level variables, was selected from the four models due to its lowest deviance, AIC, and BIC values. To evaluate multicollinearity, a variance inflation factor (VIF) analysis was performed following a pseudo-linear regression. No signs of multicollinearity were observed, as indicated by a maximum VIF value of 2.92 and an average VIF of 1.63, both of which were within acceptable limits (46).

## Results

**Figure 1:**
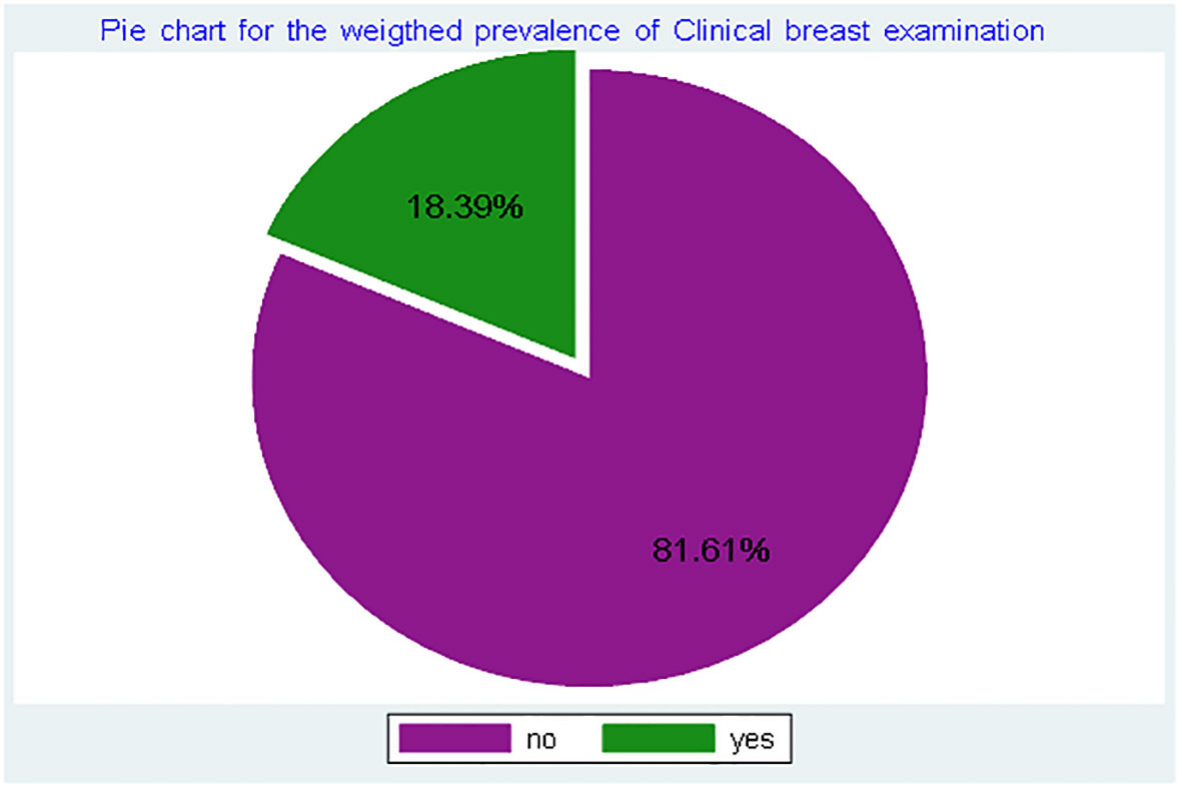
Prevalence of clinical breast examination among reproductive-age women in Ghana, using GDHS, 2022.

This study included a weighted sample of 15,013 women of reproductive age. Of these, 6,008 (40.0%) were married, and 8,998 (59.9%) had secondary education. Most respondents, 13,531 (90.1%), were covered by health insurance, while less than one-fourth, 3,639 (24.2%), used modern contraceptive methods (**Table 1**). In this study, the prevalence of CBE was 18.39% (95% CI: 17.8–19.0%) (**Figure 1**).

**Table 1:** Descriptive Characteristics of Reproductive-Aged Women in Ghana Using the GDHS, 2022.

Variables	Categories	Weighted frequency (%)	Clinical breast examination		P-value
			Yes	No	
Age	15-19	2682(17.86%)	186	2495	<0.001
	20-24	2694(17.95%)	403	2292	
	25-29	2339(15.58%)	470	1869	
	30-34	2253(15.00%)	531	1722	
	35-39	2058(13.71%)	504	1555	
	40-44	1675(11.15%)	354	1321	
	45-49	1312(8.74%)	313	999	
Marital status	Never in union	5267(35.08%)	734	4534	<0.001
	Married	6008(40.01%)	1373	4634	
	Cohabitation	2196(14.63%)	343	1853	
	Widowed	367(2.44%)	73	294	
	Divorced	389(2.59%)	87	302	
	Separated	786(5.24%)	151	635	
Level of education	No education	2411(16.06%)	220	2191	<0.001
	Primary education	2071(13.79%)	235	1836	
	Secondary education	8998(59.94%)	1581	7417	
	Diploma or above	1533(10.21%)	725	808	
Modern contraception use	Non-user	11374(75.76%)	2017	9357	<0.001
	User	3639(24.24%)	743	2896	
Covered by health insurance	No	1482(9.87%)	144	1337	<0.001
	Yes	13531(90.13%)	2616	10,916	
Parity	Null	4854(32.33%)	675	4180	<0.001
	1-3	6189(41.22%)	1412	4777	
	4 and above	3969(26.44%)	674	3295	
Current pregnancy	No	13988(93.17%)	2567	11421	**0.517**
	Yes	1025(6.83%)	194	831	
Frequency of watching television	Not at all	3463(23.07%)	376	3087	<0.001
	Less than one a week	2304(15.53%)	388	1916	
	At least once per week	9246(61.58%)	1997	7249	
Frequency of listening to radio	Not at all	4993(33.26%)	663	4330	<0.001
	Less than one a week	3674(24.47%)	621	3053	
	At least one per week	6346(42.27%)	1478	4867	
Frequency of reading newspapers or magazines	Not at all	13292(88.54%)	2251	11041	<0.001
	Less than one a week	1182(7.87%)	313	869	
	At least one per week	539(3.59%)	197	342	
Ever been tested for HIV	No	6403(42.65%)	582	5821	<0.001
	Yes	8610(57.35%)	2178	6432	
Ever tested for cervical cancer	No	14269 (95.05%)	2256	12013	<0.001
	Yes	744(4.95%)	504	240	
Currently working	Not working	3808(25.37%)	473	3335	<0.001
	Working	11205(74.63%)	2287	8918	
Visited health facility within the past 12 months	No	7225(48.13%)	933	6292	<0.001
	Yes	7788(51.78%)	1827	5961	
Sex of household head	Male	8652(57.64%)	1496	7156	<0.001
	Female	6361(42.36%)	1264	5097	
Wealth index	Poorest	2447(16.3%)	188	2259	<0.001
	Poorer	2712(18.06%)	304	2408	
	Middle	3120(20.79%)	479	2641	
	Richer	3379(22.5%)	655	2724	
	Richest	3355(22.35%)	1134	2221	
Distance to the health facility	Not a big problem	3353(22.34%)	426	2927	<0.001
	Big problem	11660(77.66%)	2335	9325	
Community level variables					
Region	Western	955(6.36%)	215	740	<0.001
	Central	1703(11.34%)	304	1399	
	Great Accra	2327(15.50%)	569	1759	
	Volta	713(4.75%)	131	582	
	Eastern	1219(8.12%)	271	949	
	Ashanti	2928(19.50%)	549	2379	
	Western north	410(2.74%)	57	353	
	Ahafo	317(2.11%)	55	262	
	Bono	567(3.78%)	108	458	
	Bono east	676(4.50%)	93	583	
	Oti	403(2.68%)	34	369	
	Northern	1148(7.65%)	177	972	
	Savannah	319(2.13%)	22	297	
	Northeast	290(1.93%)	51	239	
	Upper east	640(4.26%)	84	555	
	upper west	398(2.65%)	40	358	
Residence	Urban	8556(57.00%)	1967	6589	<0.001
	Rural	6457(43.00%)	793	5664	
Proportion of educated individuals in the community	Low	4948(32.96%)	532	4416	<0.001
	High	10,065(67.04%)	2228	7837	
Proportion of poor individuals in the community	Low	9616(64.05%)	2194	7422	<0.001
	High	5397(35.95%)	566	4831	
The proportion of individuals facing significant challenges in accessing health facilities due to distance.	Low	8452(56.30%)	1764	6688	<0.001
	High	6561(43.7%)	997	5564	
The proportion of individuals covered by health insurance in the community	Low	7929(52.81%)	1303	6626	<0.001
	High	7085(47.19%)	1458	5627	

Fixed effect analysis results: In the chi-square test except for current pregnancy status all variables have statistically significant association. Thus, variables other than current pregnancy were included in the bivariable multilevel logistic regression model. In this analysis, all variables had a p-value below 0.2, except for the sex of the household head. As a result, the sex of the household head was not considered in the multivariable mixed-effect multilevel logistic regression model. In the multivariable mixed-effects logistic regression model, several factors showed significant associations with CBE. Among the significant factors, age, level of education, modern contraceptive utilization, health insurance coverage, frequency of listening to the radio, frequency of reading newspapers or magazines, having ever been tested for HIV, having been tested for cervical cancer, current occupation, visiting a health facility within the past 12 months, wealth index, community education, and being from the Northeast region had a significant positive association with CBE. Conversely, distance to the health facility and being from the Volta, Oti, Ashanti, and Upper East regions were significantly negatively associated with CBE.

Compared to respondents aged 15-19, older respondents had higher odds of CBE (age 20-24: AOR = 1.38, 95% CI: 1.12, 1.70; age 25-29: AOR = 1.40, 95% CI: 1.11, 1.78; age 30-34: AOR = 1.65, 95% CI: 1.28, 2.12; age 35-39: AOR = 2.00, 95% CI: 1.55, 2.61; age 40-44: AOR = 1.97, 95% CI: 1.50, 2.61; and age 45-49: AOR = 2.84, 95% CI: 2.13, 3.78). Having secondary education increased the odds of CBE by 70% (AOR = 1.70, 95% CI: 1.41, 2.06) compared to having no education. Additionally, participants with diploma and above had 3.63 times higher odds of CBE (AOR = 3.63, 95% CI: 2.86, 4.61) compared to those with no education. Modern contraceptive users had 12% higher odds of CBE (AOR = 1.12, 95% CI: 1.00, 1.25) compared to non-users. Being covered by health insurance increased the odds of CBE by 53% (AOR = 1.53, 95% CI: 1.24, 1.89). Listening to the radio at least once per week increased the odds of CBE by 35% (AOR = 1.35, 95% CI: 1.20, 1.53) compared to not listening at all. Furthermore, reading a newspaper or magazine less than once per week increased the odds of CBE by 29% (AOR = 1.29, 95% CI: 1.09, 1.53), and reading a newspaper at least once per week increased the odds of CBE by 75% (AOR = 1.75, 95% CI: 1.39, 2.21). Being tested for HIV increased the odds of CBE by 92% (AOR = 1.92, 95% CI: 1.68, 2.19) compared to their counterparts. Being screened for cervical cancer increased the odds of CBE by 6.64 times (AOR = 6.64, 95% CI: 5.51, 7.99). Being currently employed increased the odds of CBE by 17% (AOR = 1.17, 95% CI: 1.02, 1.34) compared to those not working. Visiting a health facility within the past 12 months increased the odds of CBE by 36% (AOR = 1.36, 95% CI: 1.23, 1.51), controlling for other factors constant. Being from the richest wealth category increased the odds of CBE by 70% (AOR = 1.70, 95% CI: 1.27, 2.28) compared to the poorest wealth category. Participants from the Northeast region had 96% higher odds of CBE compared to the Western region (AOR = 1.96, 95% CI: 1.19, 3.22). Individuals with significant problems accessing health facilities due to distance had 24% lower odds of CBE compared to those without such issues (AOR = 0.86, 95% CI: 0.75, 0.98). Being from the Volta region decreased the odds of CBE by 36% (AOR = 0.66, 95% CI: 0.45, 0.97). Similarly, being from the Ashanti region decreased the odds of CBE by 38% (AOR = 0.62, 95% CI: 0.45, 0.85). Participants from the Oti region had 44% lower odds of CBE (AOR = 0.54, 95% CI: 0.34, 0.92), and participants from the Upper East region had 40% lower odds of CBE compared to those from the Western region. This implies that participants from the Volta, Ashanti, Oti, and Upper East regions are less likely to undertake CBE compared to those from the Western region. Participants from areas with a higher proportion of educated individuals had 31% higher odds of CBE compared to those from areas with a lower proportion of educated individuals (AOR = 1.31, 95% CI: 1.07, 1.61) (**Table 2**).

**Table 2:** Factors associated with clinical breast examination among reproductive-age women in Ghana using GDHS, 2022.

Variables	Categories	Null model	Individual level model	Community level model	A model containing individual and community-level variables
			AOR (95%CI)	AOR (95%CI)	AOR (95%)
Age	15-19		1.00		1.00
	20-24		1.42 (1/15, 1.75)		1.38 (1.12, 1.70)*
	25-29		1.44 (1.14, 1.84)		1.40 (1.11, 1.78)*
	30-34		1.69 (1.32, 2.17)		1.65 (1.28, 2.12)**
	35-39		2.02 (1.55, 2.62)		2.00 (1.55, 2.61)**
	40-44		1.94 (1.47, 2.56)		1.97 (1.50, 2.61)**
	45-49		2.84 (2.13, 3.77)		2.84 (2.13, 3.78)**
Marital status	Never in union		1.00		1.00
	Married		1.15 (0.97, 1.27)		1.10 (0.93, 1.32)
	Cohabitation		0.95 (0.78, 1.14)		0.92 (0.76, 1.12)
	Widowed		1.07 (0.76, 1.50)		1.04 (0.74, 1.47)
	Divorced		0.97 (0.71, 1.33)		0.94 (0.69, 1.30)
	Separated		1.01 (0.79, 1.30)		0.99 (0.77,1.30)
Level of education	No education		1.00		1.00
	Primary education		1.18 (0.95, 1.47)		1.18 (0.95, 1.48)
	Secondary education		1.73 (1.43, 2.08)		1.70 (1.41, 2.06)**
	Diploma or above		3.74 (2.96, 4.73)		3.63 (2.86, 4.61)**
Modern contraception use	Non-user		1.00		1.00
	User		1.11 (0.99, 1.24)		1.12 (1.00, 1.25)*
Covered by health insurance	No		1.00		1.00
	Yes		1.60 (1.3o, 1.97)		1.53 (1.24, 1.89)**
Parity	Null		1.00		1.00
	1-3		1.01 (0.85, 1.20)		1.00 (0.84, 1.19)
	4 and above		0.82 (0.65, 1.02)		0.84 (0.65, 1.02)
Frequency of watching television	Not at all		1.00		1.00
	Less than one a week		0.93 (0.77, 1.13)		0.94 (0.78, 1.13)
	At least once per week		0.94 (0.80, 1.11)		0.93 (0.79, 1.09)
Frequency of listening to radio	Not at all		1.00		1.00
	Less than one a week		1.03 (0.90, 1.19)		1.02 (0.78, 1.13)
	At least one per week		1.35 (0.80, 1.11)		1.35 (1.20, 1.53)**
Frequency of reading newspapers or magazines	Not at all		1.00		1.00
	Less than one a week		1.31 (1.11, 1.56)		1.29 (1.09, 1.53)**
	At least one per week		1.77 (1.41, 2.23)		1.75 (1.39, 2.21)**
Ever been tested for HIV.	No		1.00		1.00
	Yes		1.91 (1.67, 2.18)		1.92 (1.68, 2.19)**
Ever tested for cervical cancer	No		1.00		1.00
	Yes		6.85 (5.69, 8.25)		6.64 (5.51, 7.99)**
Currently working	Not working		1.00		1.00
	Working		1.15 (1.00, 1.31)		1.17 (1.02, 1.34)*
Visited health facility within the past 12 months	No		1.00		1.00
	Yes		1.36 (1.23, 1.51)		1.36 (1.23, 1.51**)
Wealth index	Poorest		1.00		1.00
	Poorer		1.22 (0.97, 1.53)		1.14 (0.90, 1.44)
	Middle		1.48 (1.17, 1.87)		1.24 (0.96, 1.62)
	Richer		1.40 (1.10, 1.78)		1.13 (0.86, 1.49)
	Richest		2.17 (1.69, 2.78)		1.70 (1.27, 2.28)**
Community level variables					
Region	Western			1.00	1.00
	Central			070 (0.49, 1.00)	0.76 (0.54, 1.06)
	Great Accra			0.85 (0.61, 1.20)	0.75 (0.54, 1.03)
	Volta			0.75 (0.51, 1.11)	0.66 (0.45, 0.97)*
	Eastern			0.99 (0.70, 1.41)	0.92 (0.65, 1.29)
	Ashanti			0.68 (0.49, 0.96)	0.62 (0.45,0.85)**
	Western north			0.70 (0.45, 1.10)	0.69 (0.45, 1.08)
	Ahafo			0.99 (0.62, 1.58)	1.03 (0.65, 1.64)
	Bono			0.76 (0.50, 1.15)	0.86 (0.57, 1.28)
	Bono east			0.63 (0.41, 0.97)	0.72 (0.47, 1.09)
	Oti			0.57 (0.34, 0.94)	0.56 (0.34, 0.92)*
	Northern			0.89 (0.60, 1.43)	1.00 (0.67, 1.49)
	Savannah			0.47 (0.26, 0.85)	0.65 (0.36, 1.17)
	Northeast			1.38 (0.84, 2.28)	1.96 (1.19,3.22)**
	Upper east			0.74 (0.48, 1.13)	0.69 (0.45, 1.05)
	upper west			0.62 (0.38, 1.03)	0.60 (0.36, 0.99)*
Residence	Urban			1.00	1.00
	Rural			0.75 (0.62, 0.91)	0.90 (0.74, 1.09)
Proportion of educated individuals in the community	Low			1.00	1.00
	High			1.59 (1.30, 1.97)	1.31 (1.07, 1.61)*
Proportion of poor individuals in the community	Low			1.00	1.00
	High			0.60 (0.48, 0.75)	0.82 (0.67, 1.03)
Distance to a health facility	Not a big problem		1.00		1.00
	Big problem			0.76 (0.67, 0.87)	0.86 (0.75, 0.98)*
The proportion of individuals covered by health insurance	Low			1.00	1.00
	High			1.24 (1.05, 1.45)	1.06 (0.91, 1.24)

Single asterisk (*): The coefficient is statistically significant at the 0.05 level (p < 0.05). Double asterisks (**): The coefficient is statistically significant at the 0.01 level (p < 0.01)

### Random effect results and model comparison

The presence of significant variation in CBE between clusters is indicated by the ICC and MOR in the null model. Specifically, the highest median odds ratio (2.18) suggests that a woman from a cluster with a higher prevalence of CBE has 2.18 times higher odds of undergoing the examination compared to a woman from a cluster with a lower prevalence of CBE. The ICC in the null model indicates that 17% of the variation in CBE is due to differences between clusters. The final model, which included both individual and community variables, demonstrated the best fit with the data, as evidenced by its lowest deviance value, AIC, and BIC (**Table 3**).

**Table 3:** Random effect results and model comparison for the associated factors of CBE among reproductive-age women in Ghana using GDHS, 2022.

Random effect model	Null Model	Model-I	Model-II	Model-III
Variance	0.67	0.34	0.38	0.29
ICC	0.17	0.09	0.103	0.08
MOR	2.18	1.74	1.80	1.62
Model comparison				
Log-likelihood	-6814.7	-5832.4	-6709.6	-5802.7
Deviance	13,629.4	11664.8	13419.2	11,605.4
AIC	11633.49	11734.84	13463.14	11715.44
BIC	13648.72	12001.43	13630.71	12134.36

## Discussion

Breast cancer is a significant public health concern for women, particularly in low and middle-income countries, where CBE is considered the primary screening method. The current study aimed to determine the prevalence of CBE and identify the factors associated with it. In this particular study, it was found that the prevalence of CBE was 18.39% (95%CI: 17.8-19.0%). Various factors were identified as having a significant positive association with CBE. These factors included age, level of education, utilization of modern contraceptives, having health insurance coverage, frequency of listening to the radio, frequency of reading newspapers or magazines, previous HIV testing, cervical cancer testing, current occupation, recent visits to a health facility within the past 12 months, wealth index, community education, and belonging to the North East region. Conversely, certain factors demonstrated a significant negative relationship with CBE. These factors encompassed the distance to the health facility and belonging to the Volta region, Oti region, Ashanti region, and Upper East region.

The prevalence of CBE in this study [18.39% (95%CI: 17.8-19.0%)] was found to be higher compared to previous studies conducted in Tanzania 0.9% (31), Nigeria 9.1% (32), and Lesotho 9.73% (29). However, it was lower compared to the prevalence reported in Iran 19.1% (33), Malaysia 53.3% (28), and Kenya 45% (30). The variation in the prevalence of CBE can be attributed to several factors. Differences in healthcare infrastructure may account for some of the variation, as countries with well-established healthcare systems tend to have higher rates of CBE. Cultural norms can also contribute, as certain societies may consider breast examinations by healthcare professionals to be culturally sensitive or taboo. Additionally, variations in educational attainment, income levels, and proximity to healthcare facilities may play a role in the differing prevalence rates. The magnitude of clinical breast examination in this study was found to be below the WHO recommendations. The WHO recommends CBE every one to three years for women aged 25-39, and annual CBE for women aged 40 and above (26). Indicating that a substantial portion of the population is not receiving regular screenings as advised. Specifically, our prevalence rate is lower than the recommended frequency of CBE for both age groups. This shortfall in CBE coverage has critical implications for public health interventions in Ghana. It suggests that there are barriers to accessing or utilizing CBE services, which could lead to delayed diagnoses and poorer health outcomes for women.

Older individuals had higher odds of CBE than young individuals. This finding is consistent with the study conducted in four Sub-Saharan African Countries (35), and in Lesotho (29). This could be, because older women may perceive themselves as more susceptible to breast cancer, which motivates them to prioritize screenings. Healthcare providers may recommend regular CBE as an essential component of routine healthcare for older women. In addition, as women age, their awareness of the importance of screenings tends to increase, especially when they have personal observations of breast cancer cases among their family members, neighbors, or acquaintances.

Having a secondary education or higher was associated with higher odds of CBE compared to individuals with no education. This is consistent with the study conducted in four Sub-Saharan African countries (35), and the study conducted in Kenya (36). This might be because educated individuals are more likely to know the advantages of early screening and they might be more likely to know the risk and potential signs of breast cancer. In addition, educated individuals have better access to various sources of information, like health pieces of literature. Moreover, educated individuals are less affected by the community’s thoughts and they may convince the community about the advantages of CBE.

Individuals who had health insurance coverage showed higher odds of undergoing CBE compared to those who did not have health insurance coverage. This is in line with the study conducted in Lesotho (29), the study conducted in four Sub-Saharan African countries (35), and the study conducted in Kenya (36). Having health insurance can enable individuals to receive CBE services at a minimal or no cost. Health insurance also encourages regular checkups and screenings, promoting preventive care. Moreover, if an abnormality is detected during the CBE, health insurance coverage can be extended to cover additional diagnostic testing. This coverage ensures that individuals can undergo necessary tests without facing financial obstacles.

Individuals who frequently read a newspaper or magazine had higher odds of undergoing the examination compared to those who did not read newspapers or magazines at all, this finding is consistent with the study conducted in Lesotho (29). This could be because reading magazines and newspapers can provide individuals with information and knowledge regarding CBE, including its benefits and recommended frequency. Additionally, magazines have the potential to address misconceptions and fears associated with the screening. They may offer expert advice that can help alleviate anxiety related to breast cancer screenings.

In this study, it was found that undergoing cervical cancer screening was a strong factor associated with an increased likelihood of receiving a CBE. While there is limited research directly addressing the connection between cervical cancer screening and CBE, a potential explanation could be women who undergo cervical cancer screening may receive information from healthcare providers who also offer simultaneous screening for both breast and cervical cancer. Additionally, the process of cervical cancer screening can serve as a means to promote overall women’s health, which could result in increased awareness and participation in CBE.

Being employed at present was found to be associated with higher odds of CBE. This could be attributed to several factors. Firstly, currently working mothers may have greater financial freedom, enabling them to afford transportation to healthcare facilities where CBE is conducted. Secondly, employed mothers often have diploma or above attainment, which can contribute to their awareness of the importance of regular screenings. Additionally, working mothers may receive information about CBE from their peers in the workplace, fostering knowledge sharing and encouraging participation in screenings. Furthermore, targeted campaigns may exist to provide information specifically tailored for working mothers, facilitating their access to relevant resources and increasing their awareness of CBE.

Visiting a health facility within the past 12 months increased the odds of CBE as compared to those who did not visit a health facility within the past 12 months. This is consistent with the study conducted in Lesotho (29). This could be because these individuals have the opportunity to consult healthcare providers who offer screening services specifically for breast examination. Additionally, during their visit, they may receive information about the benefits of CBE and are more likely to pursue it. Moreover, healthcare providers may recommend and encourage their clients to undergo CBE based on their medical assessment and individual risk factors.

Individuals belonging to the wealthiest wealth categories exhibited increased odds of undergoing CBE compared to those in the poorest wealth status, this is consistent with the study conducted in Four Sub-Saharan African countries (35), a study conducted in Kenya (36), and the study conducted in Malaysia (28). The higher likelihood of wealthier individuals undergoing CBE can be explained by different things. Firstly, their financial freedom enables them to have access to transportation and affords them the means to seek medical treatment if needed. This allows them to prioritize and actively pursue screenings, such as CBE. In addition to financial freedom, health insurance coverage might have a role. Wealthier individuals are more likely to have comprehensive health insurance coverage, which can cover the costs of preventive screenings like CBE. Furthermore, their proximity to healthcare facilities can contribute to the higher odds of CBE. Wealthier individuals often reside in urban areas, which typically have better access to healthcare services. This proximity makes it easier for them to access healthcare facilities offering CBE services, thereby increasing their likelihood of undergoing the screening.

Participants from the Northeast region exhibited higher odds of undergoing CBE, whereas women from the Volta, Ashanti, and Oti regions, as well as the Upper East region, demonstrated decreased odds of CBE. This finding corresponds with the results of a study carried out in Kenya (36), which underscored the substantial association between geographical location and CBE. Likewise, a study conducted in Lesotho (29) reached a similar conclusion, highlighting the significant influence of region on CBE. The variation in CBE prevalence across different regions can be attributed to different factors. One possible explanation is the economic composition of individuals within each region. Regions with a higher proportion of economically disadvantaged individuals may have lower rates of CBE. Another explanation could be the variation in infrastructure. Regions with well-developed healthcare infrastructure, including the presence of accessible health facilities, may have a higher proportion of CBE. Additionally, differences in cultural norms across regions can play a role. Clinical breast examination may be considered a routine and accepted procedure in some regions, while in others; it may be associated with cultural taboos or stigma.

Individuals facing significant challenges related to distance from healthcare facilities experienced decreased odds of undergoing CBE. This finding aligns with studies conducted in Malaysia (28) and the United Kingdom (43). This could be because the expenses and time required to travel to a health facility can hinder individuals from prioritizing and undertaking CBE. The inconvenience of scheduling and attending appointments may be particularly challenging for individuals who live far away from the health facility, especially those with busy schedules and caregiving responsibilities. Moreover, individuals residing at a distance from health facilities may have limited access to health education and information regarding CBE.

When interpreting this study’s findings, it’s important to consider its cross-sectional nature, which limits the ability to determine causality. The identified factors influencing Clinical Breast Examination (CBE) uptake, such as age, education, and wealth, may not be causal. Additionally, the study may not be generalizable to older women, as the data was collected only from women of reproductive age. The overall prevalence may also be underestimated, as older individuals are more likely to undergo screening than younger ones. The generalizability of the study might also be affected, as participants were asked about their lifetime history of screening. Women who were screened 10 or 15 years ago may be considered as screened without fulfilling the WHO recommendation of screening every 5 years for HIV-negative and every 2 years for HIV-positive individuals. Nonetheless, the study emphasizes key public health implications. Targeted interventions are needed for underserved populations, and addressing healthcare access barriers, such as regional disparities and facility distance, is vital. Expanding health insurance coverage, promoting culturally sensitive education, and aligning policies with WHO guidelines are critical to increasing CBE uptake. Routine healthcare visits can also promote early detection through regular screenings.

## Strengths and limitations of the study

Use of Nationally Representative Data: The study’s use of a nationally representative dataset is a significant strength, ensuring that the findings are generalizable to the population of Ghanaian women of reproductive age. Advanced Analytical Methods: The application of multilevel logistic regression, which accounts for the hierarchical nature of the data, is a robust choice that enhances the reliability of the results. Comprehensive Examination of Factors: The study thoroughly examines a wide range of factors associated with CBE, providing valuable insights that can inform targeted interventions. However, it is important to acknowledge the study’s limitations. As previously mentioned in the discussion part, the cross-sectional nature of the DHS data prevents the assessment of temporal relationships, making it impossible to determine whether the outcome preceded the exposure. Consequently, the data should be interpreted with caution. Future studies utilizing prospective follow-up designs are recommended to address this limitation. Additionally, the study relied on individuals’ lifetime history of CBE, which could affect the estimates, as those who were screened a long time ago may still be classified as currently screened.

## Conclusions and recommendations

The magnitude of CBE uptake in Ghana is low. Age, education level, use of modern contraceptives, health insurance coverage, frequency of radio listening and reading newspapers or magazines, history of HIV and cervical cancer testing, current occupation, recent visits to healthcare facilities, wealth index, community education, and region were significantly associated with CBE. Based on these findings, it is recommended that public health interventions focus on addressing these significant factors to improve CBE. By doing so, it is possible to increase the likelihood of early detection of breast cancer, reduce breast cancer-related mortality, and reduce the disease’s economic impact. To achieve these objectives, interventions may include targeted educational campaigns to raise awareness about the importance of CBE. Efforts to improve access to healthcare services, such as promoting health insurance coverage and encouraging regular visits to healthcare facilities, can also help to increase the uptake of clinical breast exams. Furthermore, using media outlets like radio and print media can assist in communicating information about CBE and encourage women to undergo regular CBE.

## Data Availability

The datasets presented in this study can be found in online repositories. The names of the repository/repositories and accession number(s) can be found below: http://www.dhsprogram.com.
